# *N*-Methyl-1-oxoisoindoline-2-carboxamide monohydrate

**DOI:** 10.1107/S1600536808008362

**Published:** 2008-04-02

**Authors:** Bushra Maliha, Muhammad Ilyas Tariq, M. Nawaz Tahir, Ishtiaq Hussain, Hamid Latif Siddiqui

**Affiliations:** a University of the Punjab, Institute of Chemistry, Lahore-54590, Pakistan; b University of Sargodha, Department of Chemistry, Sargodha, Pakistan; c University of Sargodha, Department of Physics, Sargodha, Pakistan; d University of the Punjab, Institute of Chemistry, Lahore 54590, Pakistan

## Abstract

The title compound, C_10_H_10_N_2_O_2_·H_2_O, is dimerized by inversion-related inter­molecular N—H⋯O hydrogen bonding. There is an intra­molecular N—H⋯O bond, resulting in a six-membered ring. Each dimer inter­acts with other dimers through hydrogen bonding with water mol­ecules. The water mol­ecules are linked to each other in a stair-like chain, thus generating two-dimensional polymeric strips. The dimers are also linked to each other through inter­molecular C—H⋯O hydrogen bonding. There are π–π inter­actions between the aromatic and heterocyclic five-membered rings [centroid–centroid distance 3.8360 (12) Å]. C—H⋯π inter­actions also exist between CH_2_ groups and aromatic rings.

## Related literature

For related literature, see: Alberto *et al.* (1994 ▶); Berger *et al.* (1999 ▶); Cignarella *et al.* (1981 ▶); Maliha *et al.* (2008 ▶); Mancilla *et al.* (2007 ▶); Toru *et al.* (1986 ▶); Wan *et al.* (2007 ▶); Straub *et al.* (2007 ▶); Maliha *et al.* (2007 ▶).
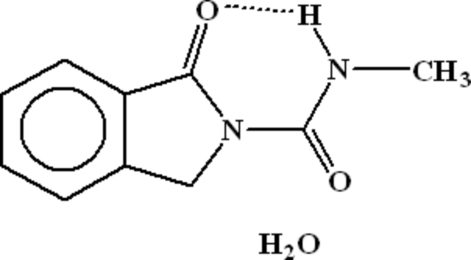


## Experimental

### 

#### Crystal data

C_10_H_10_N_2_O_2_·H_2_O
*M* *_r_* = 208.22
Monoclinic, 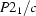

*a* = 7.4264 (4) Å
*b* = 29.0200 (16) Å
*c* = 4.8864 (2) Å
β = 108.266 (3)°
*V* = 1000.02 (9) Å^3^
*Z* = 4
Mo *K*α radiation
μ = 0.10 mm^−1^
*T* = 296 (2) K
0.22 × 0.12 × 0.10 mm

#### Data collection

Bruker Kappa APEXII CCD diffractometer
Absorption correction: multi-scan (*SADABS*; Bruker, 2005 ▶) *T*_min_ = 0.980, *T*_max_ = 0.990
18083 measured reflections
2518 independent reflections
1586 reflections with *I* > 2σ(*I*)
*R*_int_ = 0.042

#### Refinement

*R*[*F* ^2^ > 2σ(*F* ^2^)] = 0.050
*wR*(*F* ^2^) = 0.130
*S* = 1.05
2518 reflections
145 parameters
H atoms treated by a mixture of independent and constrained refinement
Δρ_max_ = 0.23 e Å^−3^
Δρ_min_ = −0.20 e Å^−3^

### 

Data collection: *APEX2* (Bruker, 2007 ▶); cell refinement: *APEX2*; data reduction: *SAINT* (Bruker, 2007 ▶); program(s) used to solve structure: *SHELXS97* (Sheldrick, 2008 ▶); program(s) used to refine structure: *SHELXL97* (Sheldrick, 2008 ▶); molecular graphics: *ORTEP-3 for Windows* (Farrugia, 1997 ▶) and *PLATON* (Spek, 2003 ▶); software used to prepare material for publication: *WinGX* (Farrugia, 1999 ▶) and *PLATON*.

## Supplementary Material

Crystal structure: contains datablocks global, I. DOI: 10.1107/S1600536808008362/bq2071sup1.cif

Structure factors: contains datablocks I. DOI: 10.1107/S1600536808008362/bq2071Isup2.hkl

Additional supplementary materials:  crystallographic information; 3D view; checkCIF report

## Figures and Tables

**Table 1:** Hydrogen-bond geometry (Å, °)

*D*—H⋯*A*	*D*—H	H⋯*A*	*D*⋯*A*	*D*—H⋯*A*
N2—H2⋯O1	0.83 (3)	2.08 (2)	2.748 (3)	137 (2)
N2—H2⋯O1^i^	0.83 (2)	2.41 (2)	3.043 (2)	134 (2)
O3—H1*W*⋯O2^ii^	0.81 (3)	2.06 (3)	2.875 (2)	177 (3)
O3—H2*W*⋯O3^iii^	0.88 (3)	1.91 (3)	2.787 (3)	174 (3)
C4—H4⋯O2^iv^	0.93	2.44	3.362 (3)	172
C8—H8*A*⋯*Cg*1^v^	0.97	2.86	3.590 (2)	133

Symmetry codes: (i) 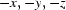; (ii) 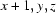; (iii) 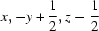; (iv) 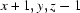; (v) 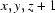. *Cg*1 is the centroid of atoms C2–C7.

**Fig. 1.**
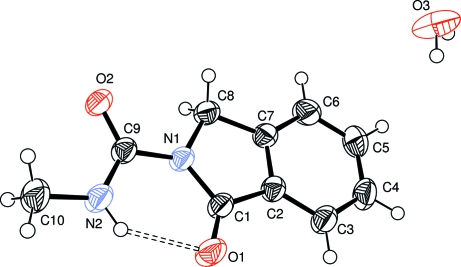
The ORTEP diagram of the title compound (I) with displacement ellipsoids at 50% probability level; intramolecular interaction has been indicated by broken line. H-atoms are shown by small circles of arbitrary radii.

**Fig. 2.**
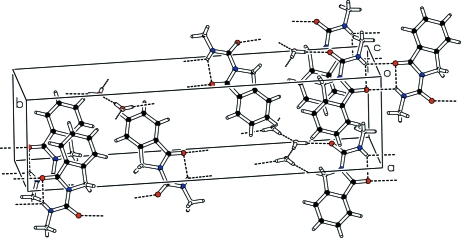
The packing figure (PLATON: Spek, 2003) which shows the H-bonding and the overlapping of rings which generate π-π interaction.

## supplementary crystallographic information

### Comment

Isoindole and their derivatives are known to be active-compounds pharmaceutically (Straub *et al.*, 2007; Mancilla *et al.*, 2007). They are important intermediates in the synthesis of novel multi-drugs resistance reversal agents (Berger *et al.*, 1999). They show diuretic, anti-anginal, cardio-vascular and herbicidal activitities (Alberto *et al.*, 1994; Cignarella *et al.*, 1981; Toru *et al.*, 1986).

The title compound (I) is in continuation to the synthesis of various isoindoles and the determination of their structures by X-ray crystallography (Maliha *et al.*,  2007; 2008). It was isolated during the studies of the reaction of urea and its *N*-alkyl/aryl derivates with *o*-phthaldehyde.

The present structure shows that replacing the H-atom of 1-Oxoisoindoline-2- carboxamide (Maliha *et al.*, 2008) with CH_3_ group, is possible in the presence of crystallization water only. The replacement of H-atom of 1-Oxoisoindoline-2-carboxamide with ethyl group (Wan *et al.*, 2007) also shows the existence of crystallizing H_2_O. Although the bond distances and bond angles in the aromatic A(C2—C7) and five-membered ring B(C1/C2/C7/C8/N1) are comparable with the reported structures (Maliha *et al.*,  2008; Wan *et al.*, 2007), but the packing through H-bonding is entirely different. There is an intramolecular hydrogen bond of N2—H2···O1 resulting in a six-membered ring. The title compound is dimerized by inversion of *N*-methyl-1-oxo-1,3-dihydro-2*H*-isoindole-2-carboxamide through intermolecular H-bond *viz* N2—H2···O1^i^ [symmetry code i = -*x*,-*y*,-*z*] and the central ring is of four members depending upon these H-bonds only. The role of H_2_O molecules is to stabilize the dimers through an interesting H-bonding. The H-bond O3—H1W···O2^ii^ [symmetry code ii = *x* + 1, *y*, *z*] connects the dimers, while the O3—H2W···O3^iii^ [symmetry code iii = *x*, -*y* + 1/2, *z* - 1/2] joints the water molecules in a stair like chain. In this way two-dimensional polymeric strip is realized. These polymeric strips are further connected by the involvement of aromatic ring A(C2—C7) through intermolecular H-bonds C4—H4···O2^iv^ [symmetry code iv = *x* + 1, *y*, *z* - 1]. The detail of H-bonding is given in Table 1 and shown in Fig. 2. The π-π interaction exist between the CgA···CgB^v^ [symmetry code v = *x*,*y*,*z* - 1] and CgB···CgA^vi^ [symmetry code vi = *x*,*y*,*z* + 1] having same centroid-centroid distance of 3.8360 (12) Å. The C—H···π interaction exists between C8—H8A and CgA^vi^ [symmetry code vi = *x*,*y*,*z* + 1] with H8A···π distance of 2.86 Å.

### Experimental

A mixture of *o*-phthaldehyde (0.67 g, 200 mmol) and *N*-methylurea (0.37 g, 200 mmol) in 100 ml of ethanol was refluxed for 10 h. The solvent was taken off and flask contents were left at room temperature. The crystals of (I) were isolated, washed with ethanol, ether and n-hexane, respectively and dried. Crystals suitable for X-ray diffraction were grown from a mixture of methanol-acetone (1:1) by slow evaporation at room temperature. It is soluble in DMSO, DMF, acetone, ethyl acetate, chloroform and carbon tetrachloride. M.P: 413 K; yield: 60 percent.

### Refinement

H atoms were positioned geometrically, with C-H = 0.93, 0.97 and 0.96 Å for aromatic, methylene and methyl C-atoms and constrained to ride on their parent atoms. The H-atoms attached to N2 and O3 atoms were located in fourier synthesis and their coordinates were refined. The thermal parameter of H-atoms of methyl group was taken 1.5 times of the parent C-atom, whereas for all other H-atoms it was taken 1.2 times of their parent atoms.

### Crystal data

**Table:**

C_10_H_10_N_2_O_2_·H_2_O	*F*_000_ = 440
*M**_r_* = 208.22	*D*_x_ = 1.383 Mg m^−^^3^
Monoclinic, *P*2_1_/*c*	Mo *K*α radiation λ = 0.71073 Å
Hall symbol: -P 2ybc	Cell parameters from 1295 reflections
*a* = 7.4264 (4) Å	θ = 1.4–28.5º
*b* = 29.0200 (16) Å	µ = 0.10 mm^−^^1^
*c* = 4.8864 (2) Å	*T* = 296 (2) K
β = 108.266 (3)º	Needle, colourless
*V* = 1000.02 (9) Å^3^	0.22 × 0.12 × 0.10 mm
*Z* = 4	

### Data collection

**Table:**

Bruker Kappa APEXII CCD diffractometer	2518 independent reflections
Radiation source: fine-focus sealed tube	1586 reflections with *I* > 2σ(*I*)
Monochromator: graphite	*R*_int_ = 0.042
Detector resolution: 7.40 pixels mm^-1^	θ_max_ = 28.5º
*T* = 296(2) K	θ_min_ = 1.4º
ω scans	*h* = −9→9
Absorption correction: multi-scan(SADABS; Bruker, 2005)	*k* = −38→38
*T*_min_ = 0.980, *T*_max_ = 0.990	*l* = −6→6
18083 measured reflections	

### Refinement

**Table:**

Refinement on *F*^2^	Secondary atom site location: difference Fourier map
Least-squares matrix: full	Hydrogen site location: inferred from neighbouring sites
*R*[*F*^2^ > 2σ(*F*^2^)] = 0.050	H atoms treated by a mixture of independent and constrained refinement
*wR*(*F*^2^) = 0.130	*w* = 1/[σ^2^(*F*_o_^2^) + (0.045*P*)^2^ + 0.3992*P*] where *P* = (*F*_o_^2^ + 2*F*_c_^2^)/3
*S* = 1.05	(Δ/σ)_max_ < 0.001
2518 reflections	Δρ_max_ = 0.23 e Å^−^^3^
145 parameters	Δρ_min_ = −0.20 e Å^−^^3^
Primary atom site location: structure-invariant direct methods	Extinction correction: none

### Special details

**Table:**

Geometry. All e.s.d.'s (except the e.s.d. in the dihedral angle between two l.s. planes) are estimated using the full covariance matrix. The cell e.s.d.'s are taken into account individually in the estimation of e.s.d.'s in distances, angles and torsion angles; correlations between e.s.d.'s in cell parameters are only used when they are defined by crystal symmetry. An approximate (isotropic) treatment of cell e.s.d.'s is used for estimating e.s.d.'s involving l.s. planes.
Refinement. Refinement of *F*^2^ against ALL reflections. The weighted *R*-factor *wR* and goodness of fit *S* are based on *F*^2^, conventional *R*-factors *R* are based on *F*, with *F* set to zero for negative *F*^2^. The threshold expression of *F*^2^ > σ(*F*^2^) is used only for calculating *R*-factors(gt) *etc*. and is not relevant to the choice of reflections for refinement. *R*-factors based on *F*^2^ are statistically about twice as large as those based on *F*, and *R*- factors based on ALL data will be even larger.

### Fractional atomic coordinates and isotropic or equivalent isotropic displacement parameters (Å^2^)

**Table:**

	*x*	*y*	*z*	*U*_iso_*/*U*_eq_	
O1	0.1330 (2)	0.04176 (5)	−0.0048 (4)	0.0582 (4)	
O2	−0.17938 (19)	0.12874 (4)	0.3549 (3)	0.0452 (4)	
O3	0.8077 (3)	0.22691 (6)	0.4262 (4)	0.0721 (6)	
H1W	0.815 (4)	0.1993 (11)	0.404 (6)	0.086*	
H2W	0.812 (4)	0.2398 (10)	0.266 (6)	0.086*	
N1	0.0204 (2)	0.11191 (5)	0.0993 (3)	0.0357 (4)	
N2	−0.1470 (3)	0.05442 (6)	0.2455 (4)	0.0477 (5)	
H2	−0.088 (3)	0.0368 (8)	0.170 (5)	0.057*	
C1	0.1337 (3)	0.08376 (6)	−0.0065 (4)	0.0377 (4)	
C2	0.2523 (3)	0.11487 (6)	−0.1134 (4)	0.0364 (4)	
C3	0.3901 (3)	0.10422 (7)	−0.2420 (4)	0.0456 (5)	
H3	0.4175	0.0738	−0.2733	0.055*	
C4	0.4848 (3)	0.14010 (8)	−0.3213 (5)	0.0498 (5)	
H4	0.5771	0.1339	−0.4083	0.060*	
C5	0.4434 (3)	0.18515 (8)	−0.2725 (5)	0.0519 (6)	
H5	0.5099	0.2089	−0.3248	0.062*	
C6	0.3054 (3)	0.19572 (7)	−0.1477 (5)	0.0487 (5)	
H6	0.2776	0.2262	−0.1178	0.058*	
C7	0.2099 (3)	0.15985 (6)	−0.0683 (4)	0.0366 (4)	
C8	0.0559 (3)	0.16101 (6)	0.0683 (4)	0.0388 (4)	
H8A	0.0976	0.1763	0.2541	0.047*	
H8B	−0.0564	0.1764	−0.0545	0.047*	
C9	−0.1088 (3)	0.09905 (6)	0.2436 (4)	0.0353 (4)	
C10	−0.2783 (3)	0.03691 (7)	0.3864 (5)	0.0572 (6)	
H10A	−0.2584	0.0044	0.4196	0.086*	
H10B	−0.4061	0.0423	0.2656	0.086*	
H10C	−0.2574	0.0524	0.5671	0.086*	

### Atomic displacement parameters (Å^2^)

**Table:**

	*U*^11^	*U*^22^	*U*^33^	*U*^12^	*U*^13^	*U*^23^
O1	0.0756 (11)	0.0306 (8)	0.0904 (12)	0.0029 (7)	0.0575 (9)	−0.0003 (7)
O2	0.0536 (8)	0.0382 (8)	0.0563 (9)	0.0004 (6)	0.0352 (7)	−0.0060 (6)
O3	0.1328 (17)	0.0403 (9)	0.0591 (10)	0.0134 (10)	0.0530 (11)	−0.0006 (8)
N1	0.0431 (9)	0.0303 (8)	0.0412 (9)	0.0006 (7)	0.0242 (7)	0.0002 (6)
N2	0.0618 (12)	0.0345 (10)	0.0632 (12)	0.0022 (8)	0.0432 (10)	0.0014 (8)
C1	0.0434 (11)	0.0340 (11)	0.0425 (10)	0.0024 (8)	0.0231 (8)	−0.0003 (8)
C2	0.0382 (10)	0.0383 (10)	0.0363 (10)	−0.0004 (8)	0.0170 (8)	0.0001 (7)
C3	0.0469 (12)	0.0447 (12)	0.0534 (12)	0.0026 (9)	0.0275 (10)	−0.0007 (9)
C4	0.0414 (12)	0.0589 (14)	0.0581 (13)	−0.0022 (10)	0.0283 (10)	0.0023 (10)
C5	0.0484 (13)	0.0526 (14)	0.0610 (14)	−0.0120 (10)	0.0260 (11)	0.0046 (10)
C6	0.0586 (14)	0.0367 (11)	0.0572 (13)	−0.0086 (10)	0.0274 (11)	−0.0032 (9)
C7	0.0404 (11)	0.0378 (10)	0.0344 (10)	−0.0026 (8)	0.0157 (8)	−0.0028 (7)
C8	0.0480 (12)	0.0307 (10)	0.0445 (11)	−0.0010 (8)	0.0245 (9)	−0.0019 (8)
C9	0.0399 (10)	0.0359 (10)	0.0347 (10)	0.0011 (8)	0.0185 (8)	0.0011 (7)
C10	0.0680 (15)	0.0460 (13)	0.0749 (16)	−0.0033 (11)	0.0475 (13)	0.0046 (11)

### Geometric parameters (Å, °)

**Table:**

O1—C1	1.219 (2)	C3—H3	0.9300
O2—C9	1.221 (2)	C4—C5	1.381 (3)
O3—H1W	0.81 (3)	C4—H4	0.9300
O3—H2W	0.88 (3)	C5—C6	1.381 (3)
N1—C1	1.384 (2)	C5—H5	0.9300
N1—C9	1.408 (2)	C6—C7	1.382 (3)
N1—C8	1.466 (2)	C6—H6	0.9300
N2—C9	1.326 (2)	C7—C8	1.495 (2)
N2—C10	1.451 (2)	C8—H8A	0.9700
N2—H2	0.83 (2)	C8—H8B	0.9700
C1—C2	1.467 (2)	C10—H10A	0.9600
C2—C7	1.377 (3)	C10—H10B	0.9600
C2—C3	1.393 (2)	C10—H10C	0.9600
C3—C4	1.378 (3)		
			
H1W—O3—H2W	106 (3)	C5—C6—C7	118.27 (19)
C1—N1—C9	128.36 (15)	C5—C6—H6	120.9
C1—N1—C8	112.66 (14)	C7—C6—H6	120.9
C9—N1—C8	118.83 (14)	C2—C7—C6	120.43 (17)
C9—N2—C10	121.47 (17)	C2—C7—C8	109.75 (15)
C9—N2—H2	117.0 (16)	C6—C7—C8	129.82 (17)
C10—N2—H2	121.4 (16)	N1—C8—C7	102.22 (14)
O1—C1—N1	125.66 (16)	N1—C8—H8A	111.3
O1—C1—C2	128.49 (16)	C7—C8—H8A	111.3
N1—C1—C2	105.84 (15)	N1—C8—H8B	111.3
C7—C2—C3	121.29 (17)	C7—C8—H8B	111.3
C7—C2—C1	109.52 (15)	H8A—C8—H8B	109.2
C3—C2—C1	129.19 (17)	O2—C9—N2	124.32 (16)
C4—C3—C2	118.08 (19)	O2—C9—N1	119.40 (16)
C4—C3—H3	121.0	N2—C9—N1	116.28 (15)
C2—C3—H3	121.0	N2—C10—H10A	109.5
C3—C4—C5	120.40 (18)	N2—C10—H10B	109.5
C3—C4—H4	119.8	H10A—C10—H10B	109.5
C5—C4—H4	119.8	N2—C10—H10C	109.5
C4—C5—C6	121.52 (19)	H10A—C10—H10C	109.5
C4—C5—H5	119.2	H10B—C10—H10C	109.5
C6—C5—H5	119.2		
			
C9—N1—C1—O1	4.1 (3)	C3—C2—C7—C8	179.20 (18)
C8—N1—C1—O1	179.5 (2)	C1—C2—C7—C8	−1.0 (2)
C9—N1—C1—C2	−175.17 (17)	C5—C6—C7—C2	0.0 (3)
C8—N1—C1—C2	0.2 (2)	C5—C6—C7—C8	−179.8 (2)
O1—C1—C2—C7	−178.7 (2)	C1—N1—C8—C7	−0.8 (2)
N1—C1—C2—C7	0.5 (2)	C9—N1—C8—C7	175.07 (15)
O1—C1—C2—C3	1.0 (4)	C2—C7—C8—N1	1.1 (2)
N1—C1—C2—C3	−179.76 (19)	C6—C7—C8—N1	−179.0 (2)
C7—C2—C3—C4	0.5 (3)	C10—N2—C9—O2	−0.6 (3)
C1—C2—C3—C4	−179.19 (19)	C10—N2—C9—N1	−179.81 (19)
C2—C3—C4—C5	0.3 (3)	C1—N1—C9—O2	170.66 (18)
C3—C4—C5—C6	−0.9 (3)	C8—N1—C9—O2	−4.5 (3)
C4—C5—C6—C7	0.8 (3)	C1—N1—C9—N2	−10.0 (3)
C3—C2—C7—C6	−0.7 (3)	C8—N1—C9—N2	174.80 (17)
C1—C2—C7—C6	179.08 (18)		

### Hydrogen-bond geometry (Å, °)

**Table:**

*D*—H···*A*	*D*—H	H···*A*	*D*···*A*	*D*—H···*A*
N2—H2···O1	0.83 (3)	2.08 (2)	2.748 (3)	137 (2)
N2—H2···O1^i^	0.83 (2)	2.41 (2)	3.043 (2)	134 (2)
O3—H1W···O2^ii^	0.81 (3)	2.06 (3)	2.875 (2)	177 (3)
O3—H2W···O3^iii^	0.88 (3)	1.91 (3)	2.787 (3)	174 (3)
C4—H4···O2^iv^	0.93	2.44	3.362 (3)	172
C8—H8A···Cg1^v^	0.97	2.86	3.590 (2)	133

Symmetry codes:  (i) −*x*, −*y*, −*z*; (ii) *x*+1, *y*, *z*; (iii) *x*, −*y*+1/2, *z*−1/2; (iv) *x*+1, *y*, *z*−1; (v) *x*, *y*, *z*+1.
